# Design and Assessment of Convolutional Neural Network Based Methods for Vitiligo Diagnosis

**DOI:** 10.3389/fmed.2021.754202

**Published:** 2021-10-18

**Authors:** Li Zhang, Suraj Mishra, Tianyu Zhang, Yue Zhang, Duo Zhang, Yalin Lv, Mingsong Lv, Nan Guan, Xiaobo Sharon Hu, Danny Ziyi Chen, Xiuping Han

**Affiliations:** ^1^Department of Dermatology, Qingdao Women and Children's Hospital of Qingdao University, Qingdao, China; ^2^Department of Computer Science and Engineering, University of Notre Dame, Notre Dame, IN, United States; ^3^Department of Computing, The Hong Kong Polytechnic University, Kowloon, Hong Kong, SAR China; ^4^Department of Dermatology, Shengjing Hospital of China Medical University, Shenyang, China; ^5^Department of Dermatology, Affiliated Central Hospital, Shenyang Medical College, Shenyang, China; ^6^Department of Dermatology, Affiliated Hospital of Medical College, Qingdao University, Qingdao, China; ^7^Department of Computer Science, City University of Hong Kong, Kowloon, Hong Kong, SAR China

**Keywords:** vitiligo, diagnosis, deep learning, machine learning, skin pigmentation

## Abstract

**Background:** Today's machine-learning based dermatologic research has largely focused on pigmented/non-pigmented lesions concerning skin cancers. However, studies on machine-learning-aided diagnosis of depigmented non-melanocytic lesions, which are more difficult to diagnose by unaided eye, are very few.

**Objective:** We aim to assess the performance of deep learning methods for diagnosing vitiligo by deploying Convolutional Neural Networks (CNNs) and comparing their diagnosis accuracy with that of human raters with different levels of experience.

**Methods:** A Chinese in-house dataset (2,876 images) and a world-wide public dataset (1,341 images) containing vitiligo and other depigmented/hypopigmented lesions were constructed. Three CNN models were trained on close-up images in both datasets. The results by the CNNs were compared with those by 14 human raters from four groups: expert raters (>10 years of experience), intermediate raters (5–10 years), dermatology residents, and general practitioners. F1 score, the area under the receiver operating characteristic curve (AUC), specificity, and sensitivity metrics were used to compare the performance of the CNNs with that of the raters.

**Results:** For the in-house dataset, CNNs achieved a comparable F1 score (mean [standard deviation]) with expert raters (0.8864 [0.005] vs. 0.8933 [0.044]) and outperformed intermediate raters (0.7603 [0.029]), dermatology residents (0.6161 [0.068]) and general practitioners (0.4964 [0.139]). For the public dataset, CNNs achieved a higher F1 score (0.9684 [0.005]) compared to the diagnosis of expert raters (0.9221 [0.031]).

**Conclusion:** Properly designed and trained CNNs are able to diagnose vitiligo without the aid of Wood's lamp images and outperform human raters in an experimental setting.

## Introduction

Vitiligo, the most common depigmentation disorder (1), can be a psychologically devastating disease that impacts the quality of life. Many dermatoses (e.g., pityriasis alba and nevus depigmentosus) may mimic vitiligo, especially at early onset. Therefore, differential diagnosis of vitiligo from other depigmented and hypopigmented lesions can be difficult (2). At present, vitiligo diagnosis is commonly accomplished by dermatologists based on patients' medical history and physical examination including inspection with Wood's lamp (3). Such a diagnosis method is largely influenced by the dermatologists' experience and subjectivity in visual perception of the depigmented skin lesions. Highly trained expert clinicians with the aid of Wood's lamps are indispensable for accurate and early detection of vitiligo, especially for patients with an atypical presentation. However, standard clinical diagnosis may fail to attain high accuracy in differentiating early vitiligo, particularly for dermatologists with less clinical experience. In the absence of Wood's light, the diagnosis accuracy could be further decreased, which hinders the development of teledermatology services since such professional equipment may not be available at the patient side (4).

Different from the diagnosis performed by human physicians that depends largely on subjective judgement and is not surely reproducible, standardized and objective deep learning (DL) tools were regarded as a potential support system able to provide reliable diagnosis of skin lesions (5). In particular, one of the common deep learning models, convolutional neural networks (CNNs), have recently shown expert-level performance in the classification of skin diseases on medical images (6–8).

Although several previous studies (9–11) have investigated CNNs for diagnosing skin disorders, prior dermatologic research involving CNNs has largely focused on pigmented/non-pigmented lesions concerning skin cancers (12–20). So far, very few studies exist on CNN-aided diagnosis of depigmented non-melanocytic lesions, such as for vitiligo which is more common but difficult to diagnose by the unaided eye (21–24). Therefore, it is under-explored whether CNNs can benefit the diagnosis of depigmented skin lesions, especially comparing to dermatologists with different levels of experience. On the other hand, many prior studies have exploited the visual recognition of skin lesions using dermoscopic images (25–28), where a dermatoscope is required. However, dermatoscopes are usually unnecessary for many kinds of common skin diseases, e.g., pigmentary issues. Thus, it is also unclear how CNNs perform if being trained with clinical photographs but without dermoscopic images.

The goal of our work is to perform a comprehensive assessment and evaluation of CNN-based techniques for vitiligo diagnosis considering various clinical scenarios. Toward this, we investigated the potential of employing CNNs for diagnosing vitiligo in the absence of highly experienced experts and Wood's lamp examination. We collected a large set of clinical close-up images with suspected vitiligo depigmentation and a public dataset through collecting a set of publicly available repositories containing vitiligo-type lesions (e.g., pityriasis alba, rosea, and versicolor) acrossing different ethnicities/races. We trained and evaluated CNNs using these images, and compared the CNNS' performance with the diagnosis conducted by dermatologists with different levels of clinical experience.

## Materials and Methods

### Deep Learning Background

In a standard DL-based process for image classification, a CNN model is first trained using a training set containing a collection of images, each image associated with a class label (29). Model training enables a CNN to take an image as input, extract the image features (abstraction), and output the final prediction as class probability. During model training, a subset of data is “held back” and periodically used for evaluating the accuracy of the model, which is called the validation set. After the model goes through the training phase utilizing the training and validation sets, a test set is used for the final evaluation to assess the performance (i.e., generalization) of the model. A test set is a collection of images that are not involved in any part of the training process and thus allows one to compare different models (or human raters) in an unbiased way.

### Datasets

**Figure 1:**
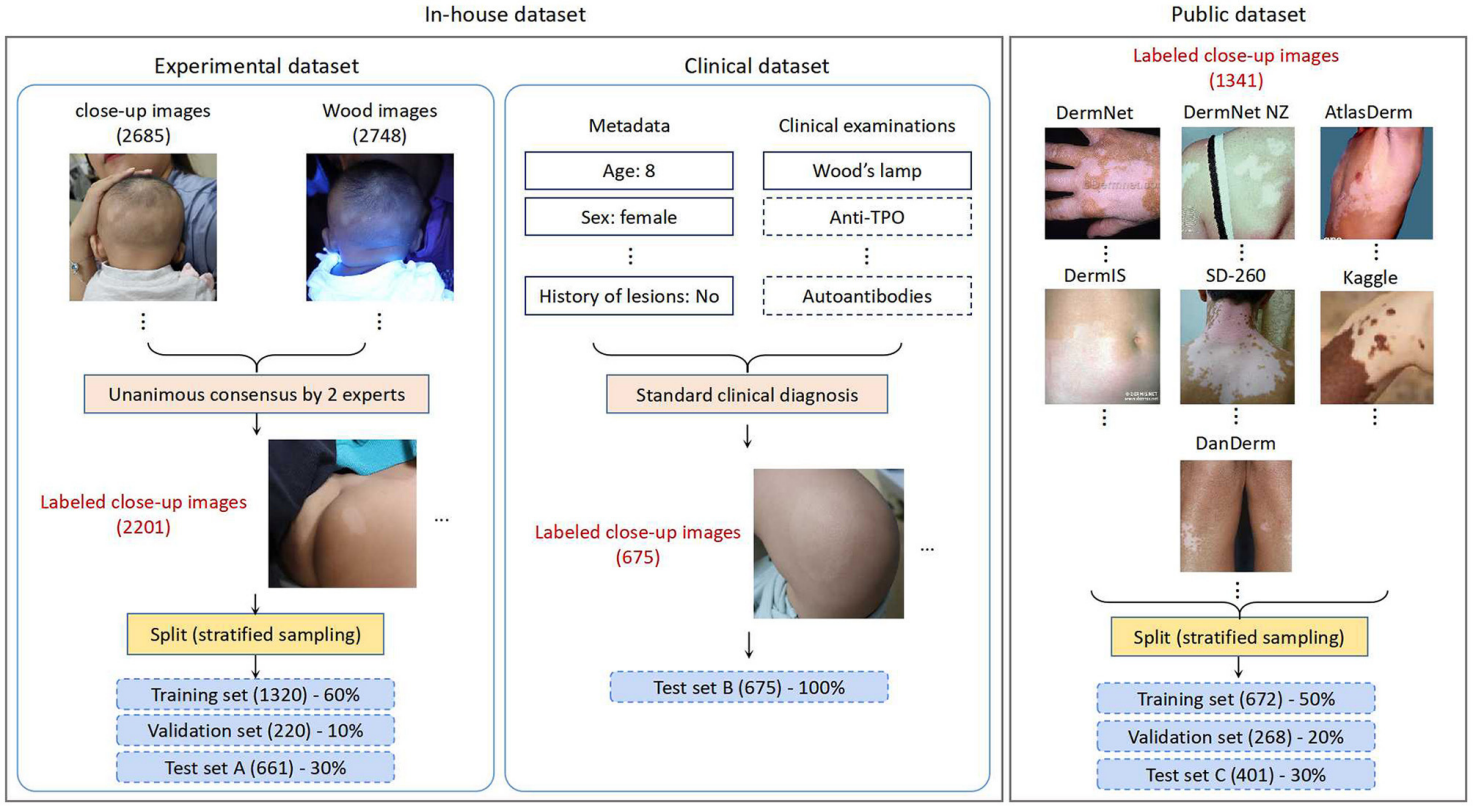
An overview of the datasets used for this study.

An in-house dataset and a public dataset were employed for this study in the various phases of CNN development (shown in Figure 1), which are discussed below.

#### In-house Dataset

The in-house dataset consists of images from retrospective consecutive outpatients obtained by the dermatology department of Qingdao Women and Children's Hospital (QWCH) in China. The data acquisition effort was approved by the institutional review board of QWCH (QFELL-YJ-2020-22 protocol). For each patient with suspected vitiligo (e.g., pityriasis alba, hypopigmented nevus), three to six clinical photographs of the affected skin areas were taken by medical assistants using a point-and-shoot camera Canon EOS 200D.

The in-house dataset was divided into two subsets based on the collection dates of the patients and the reference standard. The experimental subset contains the photographs taken from May 2019 to Dec. 2019, and was generated according to the image-based evaluation. The clinical subset contains the photographs taken from Jan. 2020 to May 2020, and was generated by dermatologists performing diagnosis in the clinical setting. Given the much larger scale, the experimental set was used throughout the CNN training, validation, and testing processes. The clinical set, on the other hand, was used as another test set for simulating a study in which a CNN model is trained on past data and tested on future cases.

Experimental Set: We extracted 1,1404 lesion images recording 1,132 patients with suspected vitiligo. Five thousand nine hundred seventy-one images with insufficient quality or duplicate lesions were excluded. The remaining 5,433 images (including 2,685 close-up and 2,748 Wood's lamp ones) from 989 patients were provided to two board-certified dermatologists with 10- and 20-years of clinical experience. The dermatologists classified these images into two classes (vitiligo, or not vitiligo) using only image-based information. Unanimous consensus was reached for 2,201 close-up images, which formed the experimental set. Following the common CNN training strategy, we performed stratified random sampling and split the experimental set into the training set (1,320 images), validation set (220 images), and test set A (661 images) with a ratio of 60:10:30.

Clinical Set: The clinical set contains 675 close-up images of 225 patients with suspected vitiligo. Each patient was evaluated through a standard clinical inspection by dermatologists, including the patient's medical history and physical examination with Wood's lamp. For patients with an atypical presentation, a blood test for checking autoimmune function was performed. Each clinical image was then labeled as vitiligo or not vitiligo according to the clinical diagnosis result. All the images in the clinical set constituted test set B, with a higher reference standard than that of test set A.

For patients with suspected vitiligo, the most common site of onset was the head and neck area (46.1%), followed by the trunk (25.3%), the limbs (23.3%), and combinations of these categories if onset occurred in multiple locations simultaneously (5.3%). The duration of the disease in our in-house dataset ranged between 0.5 and 132 months with a mean and SD of 23 ± 57 months. The level of activity for vitiligo was classified into progressive (37%), regressive (11%), or stable over the previous 6 months (52%). Most of the patients for outpatient clinic were with early onset of suspected vitiligo, and thus the lesions varied in size from 5 mm to 23 cm.

#### Public Dataset

For the sake of comprehensive performance evaluation of CNN models with an external cohort, we constructed a public dataset through collecting images of differential diagnosis of vitiligo from publicly available repositories on the Internet. We used the public dataset as a complement to the assessment of CNNs using our in-house dataset, since the public dataset contains patients of different races, ethnicities, and skin colors. The statistical data of both the public dataset and the in-house dataset is summarized in Table 1.

**Table 1:** Statistical data of the in-house dataset and public dataset.

**Dataset**		**Amount**	**Region**	**Classes**
Public	DermNet	248	US	VL & PA & TV & PB & AL & NA
	DermNet NZ	246	New Zealand	VL & ME & MO & PA & PR & PV
	AtlasDerm	147	Brazil	VL & HN & PA & PV & TV & PB & AL
	DermIS	71	Germany	VL & HN & PA & PV
	SD-260	251	Worldwide	VL & HN & PA & PV & TV
	Kaggle	368	Unknown	VL
	DanDerm	10	Denmark	VL & HN & PA
In-house		2,876	Asian	VL & HN & PA & PV & PB & AL & NA & AN

*VL, vitiligo; HN, halo nevus; PA, pityriasis alba; PV, pityriasis versicolor; PR, pityriasis rosea; ME, melasma; MO, morphea; TV, tinea versicolor; PB, piebaldism; AL, albinism; NA, nevus anemicus; AN, Achromic Nevus*.

We collected the public dataset from 7 public dermatology atlas websites: DermNet (30), DermNet NZ (31), AtlasDerm (32), DermIS (33), SD-260 (34), Kaggle (35), and DanDerm (36). Each repository contains various types of skin lesions, and we targeted skin diseases that have similar characteristics as vitiligo. The images in the integrated public dataset were divided into two classes: vitiligo (712) and not vitiligo (629), according to the classification labels in the repositories. Stratified sampling was performed to split the public dataset into the training set (50%), validation set (20%), and test set C (30%). The dataset is publicly available and can be accessed at this link.

### CNN Training Setup

We experimented with three commonly-used CNN models [VGG (37), ResNet (38), and DenseNet (39)] suitable for classification of medical images. These CNNs share a similar overall architecture consisting of two connected modules, the feature extractor module and the classifier module. The feature extractor module utilizes multiple consecutive layers of convolutions to extract a set of relevant high-level features from an input clinical image. The classifier module employs fully connected layers to generate the output as class probabilities associated with each class (vitiligo or not vitiligo). The class with highest associated probability was selected as the output class for the image.

To speed up the model training process with improved classification results, we performed transfer learning (40) that reused modules of already trained CNN models. In brief, we employed three models available in the PyTorch framework: VGG-13, ResNet-18, and DenseNet-121. These models were pre-trained with tuned network parameters using the ImageNet dataset (41). The feature extractor architecture of each network model remained unchanged while the classifier part of the model was customized for our study. In particular, the last layer of the classifier in each network model was replaced by a new layer to generate vitiligo data-specific output.

We used a standard back-propagation implementing the stochastic optimization algorithm Adam. A class balanced cross-entropy based loss function was utilized with a learning rate of 0.00002 (β1 = 0.9, β2 = 0.999, ε = 1e-8) (42). Experiments were performed on NVIDIA-TITAN and Tesla P100 GPUs using the PyTorch framework for 1000 epochs. The batch size for each experiment was selected as the maximum size allowed by the GPUs. Images were resized and normalized before training and evaluation (224 × 224). Data-augmentation operations such as horizontal and vertical flips were applied for robust feature extraction and for avoiding overfitting.

### Evaluation

The performances of the three trained CNN models on vitiligo diagnosis were evaluated using the three test sets of the in-house dataset and the public dataset, and compared with the diagnosis given by a pool of dermatologists with different levels of clinical experience.

#### Human Raters

The test participants for performance comparison with CNNs comprised of 14 human raters: four board-certified dermatologists, five dermatology residents (DRs), and five general practitioners (GPs). The board-certified dermatologists were further divided into two groups according to their years of clinical experience: two intermediate raters (5–10 years, IRs) and two expert raters (>10 years, ERs). The raters were asked to classify the clinical close-up images into vitiligo or not vitiligo.

In order to assess the diagnosis performance of the human raters in the presence or absence of Wood's lamp, the in-house dataset included two test sets in a similar scale. For test set A (661 images), corresponding Wood's lamp images (625 images in total) were provided to the human raters to aid their diagnosis, and close-up images were always shown before the corresponding Wood's lamp images. The final classification of each clinical image was based on the combination of both the imaging modalities. For test set B (675 images), only close-up images (without any Wood's lamp information) were provided to all the raters for the same classification task. For test set C (401 images), only the 2 ERs representing the highest level of clinical skills were asked to classify the close-up images in the public dataset.

#### Statistical Analysis

Using 2-tailed, paired sample *t*-tests, *p*-values were computed. For *p* < 0:05, observations were considered as statistically significant. The F1 score (F1), area under the receiver operating characteristic curve (AUC), sensitivity (SE), and specificity (SP) metrics were used for performance evaluation. Every experiment was repeated five times for variability analysis. The mean and standard deviations were used to report the outcome of each experiment.

## Results

### In-house Dataset Results

#### CNN Results

Experimental results on test set A and test set B obtained by the three CNN models are shown in Table 2. For test set A, the VGG model achieved the highest F1 scores and AUC scores. Pairwise comparison of the F1 scores revealed that the better results obtained by the VGG model (mean: 0.8864, 95% CI, 0.8913–0.8821) were statistically significant compared to the ResNet model (mean: 0.8732, 95% CI, 0.8805–0.8652; *p* = 0:030) and the DenseNet model (mean: 0.8808, 95% CI, 0.8897–0.8759; *p* = 0:011). Similar analysis for the AUC metric revealed that the better results obtained by the VGG model (mean: 0.8506, 95% CI, 0.8565–0.8437) were statistically significant in comparison to the ResNet model (mean: 0.8397, 95% CI, 0.8466–0.8300; *p* = 0:001) but not in comparison to the DenseNet model (mean: 0.8465, 95% CI, 0.8597–0.8306; *p* > 0:05). Besides, the VGG model attained the best specificity (SP = 0.9129) and sensitivity (SE = 0.7905).

**Table 2:** Quantitative results of CNN based vitiligo classification.

	**F1**	**AUC**	**SP**	**SE**
**(a) Test Set A**				
VGG	0.8864 ± 0.005	0.8506 ± 0.005	0.9129 ± 0.012	0.7905 ± 0.023
ResNet	0.8732 ± 0.006	0.8397 ± 0.007	0.9019 ± 0.014	0.7753 ± 0.025
DenseNet	0.8808 ± 0.005	0.8465 ± 0.011	0.9064 ± 0.013	0.7838 ± 0.037
**(b) Test Set B**				
VGG	0.7649 ± 0.015	0.7302 ± 0.008	0.7905 ± 0.021	0.6699 ± 0.014
ResNet	0.7363 ± 0.031	0.7024 ± 0.023	0.7614 ± 0.042	0.6434 ± 0.039
DenseNet	0.7562 ± 0.024	0.7401 ± 0.028	0.7682 ± 0.021	0.7119 ± 0.038
**(c) Test Set C**				
VGG	0.9684 ± 0.005	0.9946 ± 0.005	0.9629 ± 0.012	0.9721 ± 0.002
ResNet	0.9584 ± 0.004	0.9848 ± 0.007	0.9567 ± 0.016	0.9515 ± 0.010
DenseNet	0.9618 ± 0.030	0.9819 ± 0.027	0.9609 ± 0.027	0.9623 ± 0.032

On test set B, the VGG model achieved the best F1 scores (mean: 0.7649, 95% CI, 0.7813–0.7500). Pairwise comparison revealed that the results by the VGG model were not statistically significant compared with the ResNet model (mean: 0.7363, 95% CI, 0.7813–0.7024; *p* > 0:05) and the DenseNet model (mean: 0.7562, 95% CI, 0.7932–0.7351; *p* > 0:05). For the AUC metric, the DenseNet model (mean: 0.7401, 95% CI, 0.7819–0.7175) achieved the best scores. In comparison to the ResNet model (mean: 0.7024, 95% CI, 0.7411–0.6825; *p* = 0:036), DenseNet showed significant improvement, but not with respect to the VGG model (mean: 0.7302, 95% CI, 0.7411–0.7228; *p* > 0:05). The VGG model achieved the best specificity (SP = 0.7905), and the DenseNet model achieved the best sensitivity (SE = 0.6699).

Observe that the F1 scores for test set B were overall lower compared to test set A. This can be attributed to the differences in the datasets used for the network training and testing. Specifically, being trained and validated on one dataset, e.g., the experimental data (test set A), CNN models often suffer from the domain shift phenomenon (41) when being tested on another dataset, e.g., the clinical data (test set B), which may have certain different characteristics and features that the trained models have not seen sufficiently. Such differences between image datasets may be induced by different imaging settings (e.g., imaging equipment, equipment parameters, lighting conditions, etc., used in different clinics). A domain shift can cause a trained model to have lower performance when being tested on a dataset with somewhat (or even considerably) different characteristics, which may be the case for test set B. We speculate that a domain shift could even affect some less experienced human raters. Further inspection revealed that the drop in F1 score (and other metrics) on test set B was mainly caused by a larger number of false negative cases, i.e., a vitiligo lesion was classified by the CNNs as a not vitiligo one.

#### Comparison With Human Raters

Experimental results on test set A and test set B obtained by human raters are shown in Table 3. On test set A, the average F1 score achieved by the ERs was 0.8933 (mean: 0.8933, 95% CI, 0.9247–0.8619), which was comparable to the best F1 score obtained by the VGG model (mean: 0.8864, 95% CI, 0.8913–0.8821). The average F1 scores of the IRs (mean: 0.7603, 95% CI, 0.7806–0.7400), the DRs (mean: 0.6161, 95% CI, 0.6939–0.5231) and GPs (mean: 0.4964, 95% CI, 0.6667–0.3469) were significantly lower than those of all the CNN models. On test set B, the ERs had the highest F1 score (mean: 0.7708, 95% CI, 0.8315–0.7103) among all the human raters. IRs, DRs, and GPs achieved average F1 scores of 0.7193 (mean: 0.7193, 95% CI, 0.7339–0.7047), 0.6243 (mean: 0.6243, 95% CI, 0.7500–0.5569), and 0.4633 (mean: 0.4633, 95% CI, 0.6000–0.1363), respectively. The VGG model attained a similar F1 score as that of ERs, and significantly outperformed all the other human raters in F1 score (VGG mean: 0.7649, 95% CI, 0.7812–0.7500) on test set B. It is interesting to note that both ERs and IRs have relatively high sensitivity compared to the CNNs, indicating lower false negative cases.

**Table 3:** Quantitative results of vitiligo classification by human raters with different levels of clinical experience.

	**F1**	**SP**	**SE**
**(a) Test Set A**			
Expert Raters (ER)	0.8933 ± 0.0440	0.9989 ± 0.0020	0.8107 ± 0.0750
Intermediate Raters (IR)	0.7603 ± 0.0290	0.7615 ± 0.0020	0.9369 ± 0.0550
Dermatology Residents (DR)	0.6161 ± 0.0680	0.7387 ± 0.1130	0.7840 ± 0.1040
General Practitioners (GP)	0.4964 ± 0.1390	0.5013 ± 0.3194	0.7520 ± 0.0910
**(b) Test Set B**			
Expert Raters (ER)	0.7708 ± 0.0850	0.6465 ± 0.2070	0.9375 ± 0.0170
Intermediate Raters (IR)	0.7193 ± 0.0210	0.5086 ± 0.0120	0.9625 ± 0.0530
Dermatology Residents (DR)	0.6243 ± 0.0740	0.6117 ± 0.2210	0.7050 ± 0.1580
General Practitioners (GP)	0.4633 ± 0.1910	0.6275 ± 0.2640	0.5200 ± 0.2830
**(c) Test Set C**			
Expert Raters (ER)	0.9221 ± 0.0310	0.8028 ± 0.0570	0.9642 ± 0.0280

#### Analysis of Wrongly Predicted Cases

The differential diagnoses leading to wrongly classified cases by the human raters include pityriasis alba, achromic nevus, piebaldism, and pityriasis versicolor. Skin lesions with white patches/macules and clear boundaries were easily misdiagnosed as vitiligo (see examples in the Supplementary Material), and only very few vitiligo cases were misclassified as non-vitiligo due to light patch color. Thus, human raters demonstrated relatively high sensitivity. The factors causing wrong predictions can be (i) the color difference between patient skin and lesion, (ii) the photo lighting, and (iii) the shape and boundary of lesions. For CNNs with respect to black box predictors, no distinctive features were observed among all the wrongly classified cases.

### Public Dataset Results

Table 2(c) shows the experimental results obtained by the CNNs on the public dataset (test set C). The VGG model (F1–mean: 0.9684, 95% CI, 0.9726–0.9626; AUC–mean: 0.9946, 95% CI, 0.9918–0.9961) outperformed the other two CNNs in both F1 score and AUC ([ResNet] F1 mean: 0.9584, 95% CI, 0.9626–0.9551; AUC mean: 0.9848, 95% CI, 0.9915–0.9768; [DenseNet] F1 mean: 0.9618, 95% CI, 0.9825–0.9277; AUC mean: 0.9819, 95% CI, 0.9993–0.9511). However, the improvement shown by the VGG model was not statistically significant (*p* > 0:05) compared to the other two CNN models. In comparison to the ERs, the VGG model achieved better accuracy in all the metrics and the reason may be that CNNs were trained using a portion of the public dataset while human raters were not. Such high accuracy highlights the effectiveness of the CNNs in discriminating between vitiligo and not vitiligo skin lesions.

## Discussion

Vitiligo is a psychologically devastating skin disorder as it typically occurs in exposed areas (the face and hands) and has a major impact on self-esteem. In the new media era, people's awareness of vitiligo has increased rapidly. This in turn has led to an increasing number of people seeking for vitiligo diagnosis in hospitals. On the other hand, the successes of CNNs in medical image classification applications have brought excitement in recent years. In this context, we aimed to develop and train CNNs to diagnose vitiligo with an accuracy comparable to human raters by using only clinical photographs. This enables potential teledermatology with remote diagnosis services to reduce the reliance on common clinical medical resources to a certain extent, especially in the context of the current epidemic.

In order to simulate vitiligo diagnosis in real telemedicine scenarios, we have used a large and balanced dataset of clinical images taken by a camera, instead of dermoscopic images acquired from dermatoscopes. Although dermoscropic photographs are able to capture accurate details of perilesional skin lesions (e.g., the starburst appearance, comet tail appearance), and thus ease the differentiation of vitiligo lesions from other visually similar hypopigmentary disorders, dermatoscopes are usually unnecessary for pigmentary issues in clinical settings and dermoscope is not even available in many dermatology departments. Furthermore, our in-house dataset contained images capturing lesions with depigmented skin or white patches/macules, which can be used for differential diagnosis of vitiligo. This is markedly different from the known DL-based vitiligo classifications in the literature where only normal-looking pigmented skin (22, 24) or vascular tumors (23) were selected as the non-vitiligo class.

Wood's lamp is a common diagnostic tool in dermatology. On vitiligo, due to the loss of epidermal melanin, depigmented patches appear bright bluish-white with sharp demarcations in Wood's light, thus making Wood's lamp quite useful for the diagnosis of vitiligo. In this study, Wood's lamp images were offered to aid the diagnosis of the human raters in a certain test set (test set A), while the CNNs used only clinical images for both training and testing. This provided a distinct advantage to the human raters on the classification task of test set A. The reasons why CNNs not using Wood's lamp images were two-fold. First, CNN training using both close-up images and Wood's lamp images requires that the image acquisition for both these two types of images captures exactly the regions of the same lesions with well-aligned one-to-one correspondence, which is infeasible in practice. Further, new CNN models must be developed for multi-modal image classification for vitiligo diagnosis using both close-up and Wood's lamp images, which are currently not known in the literature. Second, although Wood's lamp itself is quite inexpensive and quite common in hospitals, such equipment may not be available at the patient side in teledermatology scenarios and its effective use requires professional training.

We performed three-fold evaluations on the diagnostic ability of dermatologists in situations where only image data were available while face-to-face clinical examination was not possible. First, during the generation of the experimental dataset, 484 (18.03%) images were classified into mutually disagreeing results by the two expert dermatologists. This demonstrated that even board-certified experts cannot make highly accurate vitiligo diagnosis using only image-based information (i.e., clinical and Wood's lamp images). This was further confirmed by the quantitative results of vitiligo classification by another two experts on test set A for which the average F1 score was 0.8933. Second, the involvement of human raters with different levels of experience in our evaluation demonstrated that vitiligo diagnosis is largely influenced by the dermatologists' experience and their subjectivity, as a clear diagnostic performance difference was observed among human raters with different clinical experience for both test set A and test set B. Third, a horizontal comparison for each human rater group between test set A and test set B solidified the importance of using Wood's lamp in clinical examinations. Specifically, in the absence of Wood's lamp information in test set B, the overall accuracy of the intermediate raters decreased significantly.

The possibility of deploying CNN models for vitiligo diagnosis was assessed in two aspects. On the one hand, in comparison with human raters with different clinical experience, CNNs outperformed all the dermatologists (except the ERs) when only clinical images were provided. In the presence of Wood's lamp information to human raters, CNNs achieved comparable accuracy with that of the ERs and outperformed all the human raters with less experience. On the other hand, the high accuracy achieved on the public dataset validated the capability of our trained CNN models on external cohort. The much higher F1 score compared to that for the in-house dataset was possibly due to the facts that (i) most of the images in the public dataset capture very typical vitiligo lesions, and (ii) vitiligo in dark skinned individuals is more easily diagnosed (3). This observation was consistent for the ERs who achieved a notable accuracy improvement on test set C over test set B. The performance difference among CNN models confirmed that (i) a CNN should be carefully designed to maximize the performance, (ii) transfer learning is quite helpful in dealing with small training datasets in medical applications, and (iii) it would be beneficial to consider domain shifts (43) in CNN training.

There are still several limitations associated with our study. First, for our in-house dataset, patients were all ethnically Asian women and children. Different ethnicities/races will be incorporated in our future works which may further improve the vitiligo diagnosis ability of CNNs. Second, this study was restricted to pure image-based information and we did not include non-image information such as age, gender, and history of the lesions (44, 45). Multi-modal data based investigation could be explored as metadata is commonly available which may be used as part of the input to teledermatology services. Third, we adopted clinical evaluation results by expert dermatologists for data annotation, instead of using histological examinations. This is because it is rarely necessary to perform a skin biopsy to confirm a diagnosis in current clinical practice (1).

In conclusion, our findings suggest the potential benefits of deep learning methods as a remote diagnostic technique for vitiligo in telemedicine scenarios where Wood's lamp is not available. We think that the CNN method assessed in this work is able to play an assistant role in the teledermatology setting, while the final diagnosis decision should still be made by expert dermatologists whenever possible. For example, patients may upload skin lesion images taken using their smartphones after which the doctors can determine whether an outpatient examination is needed based on the CNN output and the patients' metadata. Further research is needed to evaluate the models' performance on individuals of different races and ethnicities. As future work, we will explore the possibility of using CNN models to evaluate the activity of skin lesions which may significantly benefit the consequent therapeutic treatment.

## Data Availability Statement

The datasets for this study can be found at https://doi.org/10.6084/m9.figshare.15067257.v1.

## Ethics Statement

The studies involving human participants were reviewed and approved by Institutional Review Board of Qingdao Women and Children's Hospital. Written informed consent to participate in this study was provided by the participants' legal guardian/next of kin.

## Author Contributions

LZ and SM: had full access to the data, take responsibility for the integrity of the data and accuracy of the data analysis, and statistical analysis. LZ, SM, TZ, ML, and NG: study concept and design. SM: developed the deep learning algorithm. LZ, YZ, DZ, and YL: acquisition of data. SM, XHu, and DC: analysis and interpretation of data. LZ, SM, XHu, DC, and XHa: drafting of manuscript. XHu, DC, and XHa: study supervision. All authors contributed to the article and approved the submitted version.

## Funding

The research of SM and DC was supported in part by US National Science Foundation [grant number CCF-1617735].

## Conflict of Interest

The authors declare that the research was conducted in the absence of any commercial or financial relationships that could be construed as a potential conflict of interest.

## Publisher's Note

All claims expressed in this article are solely those of the authors and do not necessarily represent those of their affiliated organizations, or those of the publisher, the editors and the reviewers. Any product that may be evaluated in this article, or claim that may be made by its manufacturer, is not guaranteed or endorsed by the publisher.

## References

[1] Taïeb A Picardo M. Vitiligo. N Engl J Med. (2009) 360:160–9. 10.1056/NEJMcp0804388 19129529

[2] Goh BK Pandya AG. Presentations, signs of activity, and differential diagnosis of vitiligo. Dermatol Clin. (2017) 35:135–44. 10.1016/j.det.2016.11.004 28317523

[3] Alikhan A Felsten LM Daly M Petronic-Rosic V. Vitiligo: a comprehensive overview: part I. Introduction, epidemiology, quality of life, diagnosis, differential diagnosis, associations, histopathology, etiology, and work-up. J Am Acad Dermatol. (2011) 65:473–91. 10.1016/j.jaad.2010.11.061 21839315

[4] Gawkrodger DJ Ormerod AD Shaw L Mauri-Sole I Whitton ME Watts MJ. Guideline for the diagnosis and management of vitiligo. Br J Dermatol. (2008) 159:1051–76. 10.1111/j.1365-2133.2008.08881.x 19036036

[5] Tognetti L Bonechi S Andreini P Bianchini M Scarselli F Cevenini G. A new deep learning approach integrated with clinical data for the dermoscopic differentiation of early melanomas from atypical nevi. J Dermatol Sci. (2021) 101:115–22. 10.1016/j.jdermsci.2020.11.009 33358096

[6] Esteva A Kuprel B Novoa RA Ko J Swetter SM. Dermatologist-level classification of skin cancer with deep neural networks. Nature. (2017) 542:115–8. 10.1038/nature21056 28117445 PMC8382232

[7] Han SS Kim MS Lim W Park GH Park I Chang SE. Classification of the clinical images for benign and malignant cutaneous tumors using a deep learning algorithm. J Investig Dermatol. (2018) 138:1529–38. 10.1016/j.jid.2018.01.028 29428356

[8] Tschandl P Kittler H Argenziano G. A pretrained neural network shows similar diagnostic accuracy to medical students in categorizing dermatoscopic images after comparable training conditions. Br J Dermatol. (2017) 177:867–9. 10.1111/bjd.15695 28569993

[9] Tiwari P Colborn KL Smith DE Xing F Ghosh D Rosenberg MA. Assessment of a machine learning model applied to harmonized electronic health record data for the prediction of incident atrial fibrillation. JAMA Netw Open. (2020) 3:e1919396. 10.1001/jamanetworkopen.2019.19396 31951272 PMC6991266

[10] Cullell-Dalmau M Otero-Viñas M Manzo C. Research techniques made simple: deep learning for the classification of dermatological images. J Investig Dermatol. (2020) 140:507–14. 10.1016/j.jid.2019.12.029 32087827

[11] Fujisawa Y Inoue S Nakamura Y. The possibility of deep learning-based, computer-aided skin tumor classifiers. Front Med. (2019) 6:191. 10.3389/fmed.2019.00191 31508420 PMC6719629

[12] Novoa RA Gevaert O Ko JM. Marking the path toward artificial intelligence–based image classification in dermatology. JAMA Dermatol. (2019) 155:1105–6. 10.1001/jamadermatol.2019.1633 31411643

[13] Young AT Xiong M Pfau J Keiser MJ Wei ML. Artificial intelligence in dermatology: a primer. J Investig Dermatol. (2020) 140:1504–12. 10.1016/j.jid.2020.02.026 32229141

[14] Tschandl P Rosendahl C Akay BN Argenziano G Blum A Braun RP. Expert-level diagnosis of nonpigmented skin cancer by combined convolutional neural networks. JAMA Dermatol. (2019) 155:58–65. 10.1001/jamadermatol.2018.4378 30484822 PMC6439580

[15] Fujisawa Y Otomo Y Ogata Y Nakamura Y Fujita R Ishitsuka Y. Deep-learning-based, computer-aided classifier developed with a small dataset of clinical images surpasses board-certified dermatologists in skin tumour diagnosis. Br J Dermatol. (2019) 180:373–81. 10.1111/bjd.16924 29953582

[16] Cho SI Sun S Mun JH Kim C Kim SY Cho S. Dermatologist-level classification of malignant lip diseases using a deep convolutional neural network. Br J Dermatol. (2020) 182:1388–94. 10.1111/bjd.18459 31449661

[17] Sies K Winkler JK Fink C Bardehle F Toberer F Buhl T. Past and present of computer-assisted dermoscopic diagnosis: performance of a conventional image analyser versus a convolutional neural network in a prospective data set of 1,981 skin lesions. Eur J Cancer. (2020) 135:39–46. 10.1016/j.ejca.2020.04.043 32534243

[18] Chung Y van Der Sande AA de Roos KP Bekkenk MW de Haas ER. Poor agreement between the automated risk assessment of a smartphone application for skin cancer detection and the rating by dermatologists. J Eur Acad Dermatol Venereol. (2020) 34:274–8. 10.1111/jdv.15873 31423673 PMC7027514

[19] Bejnordi BE Veta M Van Diest PJ Van Ginneken B Karssemeijer N Litjens G. Diagnostic assessment of deep learning algorithms for detection of lymph node metastases in women with breast cancer. Jama. (2017) 318:2199–210. 10.1001/jama.2017.14585 29234806 PMC5820737

[20] Han SS Moon IJ Lim W Suh IS Lee SY Na JI. Keratinocytic skin cancer detection on the face using region-based convolutional neural network. JAMA Dermatol. (2020) 156:29–37. 10.1001/jamadermatol.2019.3807 31799995 PMC6902187

[21] Liu Y Jain A Eng C Way DH Lee K Bui P. A deep learning system for differential diagnosis of skin diseases. Nat Med. (2020) 26:900–8. 10.1038/s41591-020-0842-3 32424212

[22] Liu J Yan J Chen J Sun G Luo W. Classification of vitiligo based on convolutional neural network. In: Sun X Pan Z Bertino E editors. International Conference on Artificial Intelligence and Security 2019 Jul 26. Cham: Springer (2019), 214–23.

[23] Nosseir A Shawky MA. Automatic classifier for skin disease using k-NN and SVM. in Proceedings of the 2019 8th international conference on software and information engineering 2019 Apr 9. Cairo. p. 259–62.

[24] Luo W Liu J Huang Y Zhao N. An effective vitiligo intelligent classification system. J Ambient Intell Humaniz Comput. (2020) 1–10. 10.1007/s12652-020-02357-5

[25] Yuan Y Chao M Lo YC. Automatic skin lesion segmentation using deep fully convolutional networks with jaccard distance. IEEE Trans Med Imaging. (2017) 36:1876–86. 10.1109/TMI.2017.2695227 28436853

[26] Haenssle HA Fink C Schneiderbauer R Toberer F Buhl T Blum A. Man against machine: diagnostic performance of a deep learning convolutional neural network for dermoscopic melanoma recognition in comparison to 58 dermatologists. Ann Oncol. (2018) 29:1836–42. 10.1093/annonc/mdy166 29846502

[27] Brinker TJ Hekler A Enk AH Klode J Hauschild A Berking C. Deep learning outperformed 136 of 157 dermatologists in a head-to-head dermoscopic melanoma image classification task. Eur J Cancer. (2019) 113:47–54. 10.1016/j.ejca.2019.04.001 30981091

[28] Yap J Yolland W Tschandl P. Multimodal skin lesion classification using deep learning. Exp Dermatol. (2018) 27:1261–7. 10.1111/exd.13777 30187575

[29] Du-Harpur X Watt FM Luscombe NM Lynch MD. What is AI? applications of artificial intelligence to dermatology. Br J Dermatol. (2020) 183:423–30. 10.1111/bjd.18880 31960407 PMC7497072

[30] DermNet [Internet]. Available online at: http://www.dermnet.com/ (accessed July 19, 2021).

[31] DermNetNZ [Internet].Available online at: https://www.dermnetnz.org/ (accessed July 19, 2021).

[32] AtlasDerm [Internet]. Available online at: http://www.atlasdermatologico.com.br/ (accessed July 19, 2021).

[33] DermIS [Internet]. Available online at: https://www.dermis.net/ (accessed July 19, 2021).

[34] SD-260 [Internet]. Available online at: http://xiaopingwu.cn/assets/projects/sd-198/ (accessed July 19, 2021).

[35] Kaggle [Internet]. Available online at: https://www.kaggle.com/shaikhshahid/vitiligo-images (accessed July 19, 2021).

[36] DanDerm [Internet]. Available online at: http://www.danderm.dk/ (accessed July 19, 2021).

[37] Simonyan K Zisserman A. Very deep convolutional networks for large-scale image recognition. arXiv [Preprint] arXiv:1409.1556. (2014).

[38] He K Zhang X Ren S Sun J. Deep residual learning for image recognition. In: Proceedings of the IEEE Conference on Computer Vision and Pattern Recognition. Las Vegas, NV (2016). p. 770–8.

[39] Huang G Liu Z Van Der Maaten L Weinberger KQ. Densely connected convolutional networks. In Proceedings of the IEEE Conference on Computer Vision and Pattern Recognition. Honolulu, HI (2017) p. 4700–8. 10.1109/CVPR.2017.243

[40] Shin H C Roth H R Gao M Lu L Xu Z Nogues I. Deep convolutional neural networks for computer-aided detection: CNN architectures, dataset characteristics and transfer learning. IEEE Trans Med Imaging. (2016) 35:1285–98. 10.1109/TMI.2016.2528162 26886976 PMC4890616

[41] Deng J Dong W Socher R Li LJ Li K Fei-Fei L. Imagenet: a large-scale hierarchical image database. In 2009 IEEE conference on Computer Vision and Pattern Recognition. Miami, FL: IEEE (2009). p. 248–55.

[42] Mishra S Chen D Z Hu X S. A data-aware deep supervised method for retinal vessel segmentation. In: 2020 IEEE 17th International Symposium on Biomedical Imaging (ISBI). Iowa City, IA: IEEE (2020). p. 1254–7.

[43] Stacke K Eilertsen G Unger J Lundström C. A closer look at domain shift for deep learning in histopathology. arXiv [Preprint] arXiv:1909.11575. (2019). 10.1109/JBHI.2020.3032060 33085623

[44] Kharazmi P Kalia S Lui H Wang ZJ Lee TK. A feature fusion system for basal cell carcinoma detection through data-driven feature learning and patient profile. Skin Res Technol. (2018) 24:256–64. 10.1111/srt.12422 29057507

[45] Chin YP Hou ZY Lee MY Chu HM Wang HH Lin YT. A patient-oriented, general-practitioner-level, deep-learning-based cutaneous pigmented lesion risk classifier on a smartphone. Br J Dermatol. (2020) 182:1498–500. 10.1111/bjd.18859 31907926

