# Supplementary material for: Design and Assessment of Convolutional Neural Network Based Methods for Vitiligo Diagnosis
Source: Front Med (Lausanne). 2021 Oct 18;8:754202. doi: 10.3389/fmed.2021.754202 (PMC8558218; doi:10.3389/fmed.2021.754202)

Supplementary Material

Example images that were wrongly classified by human raters.

1. A pityriasis alba case wrongly classified as vitiligo by 12 out of 14 human raters.

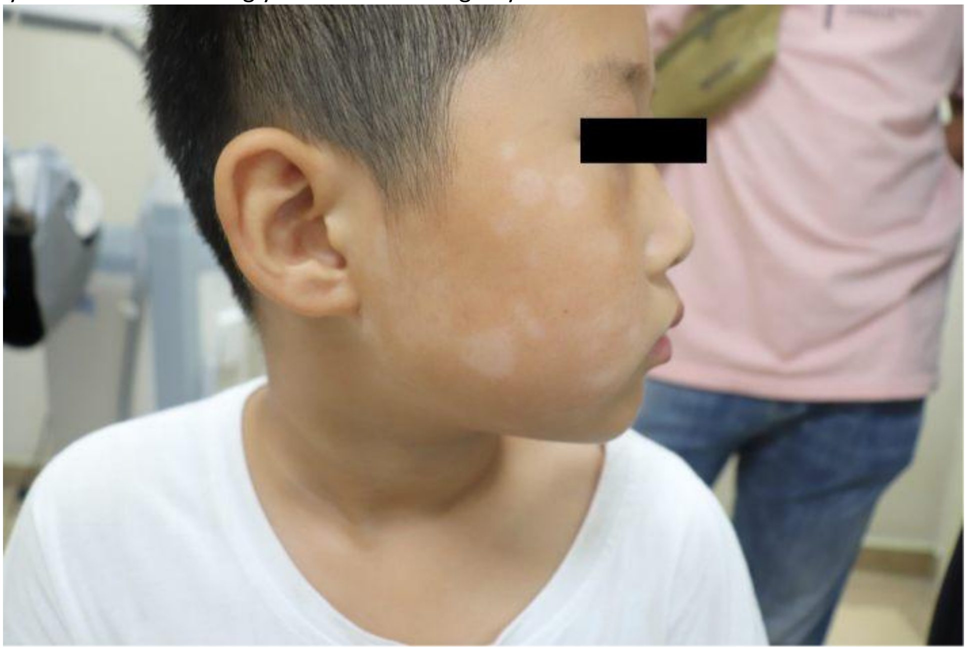

2. A pityriasis alba case wrongly classified as vitiligo by 11 out of 14 human raters.

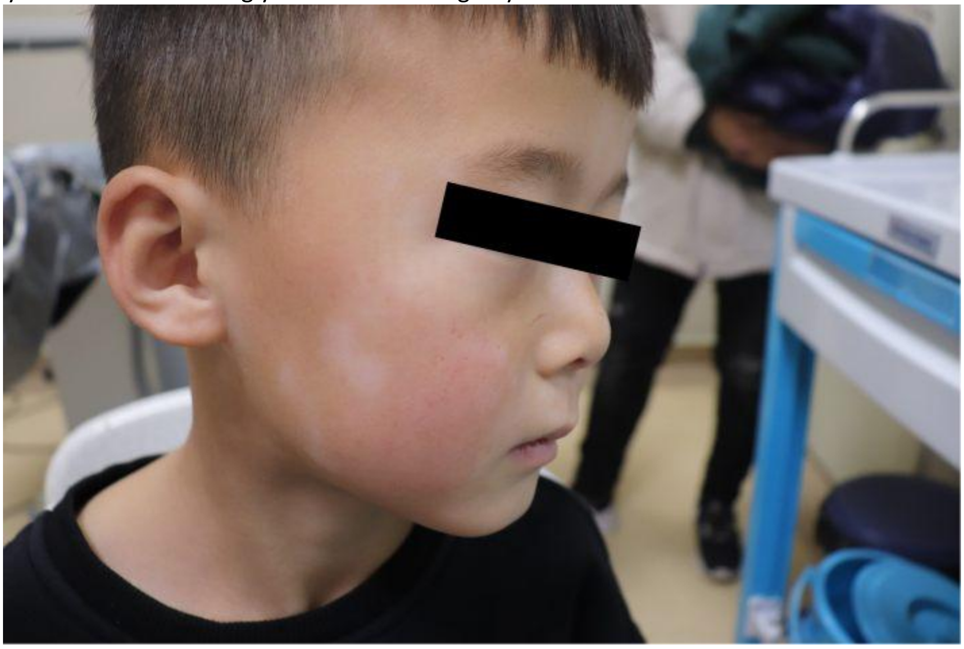

3. A nevus anemicus case wrongly classified as vitiligo by 12 out of 14 human raters.

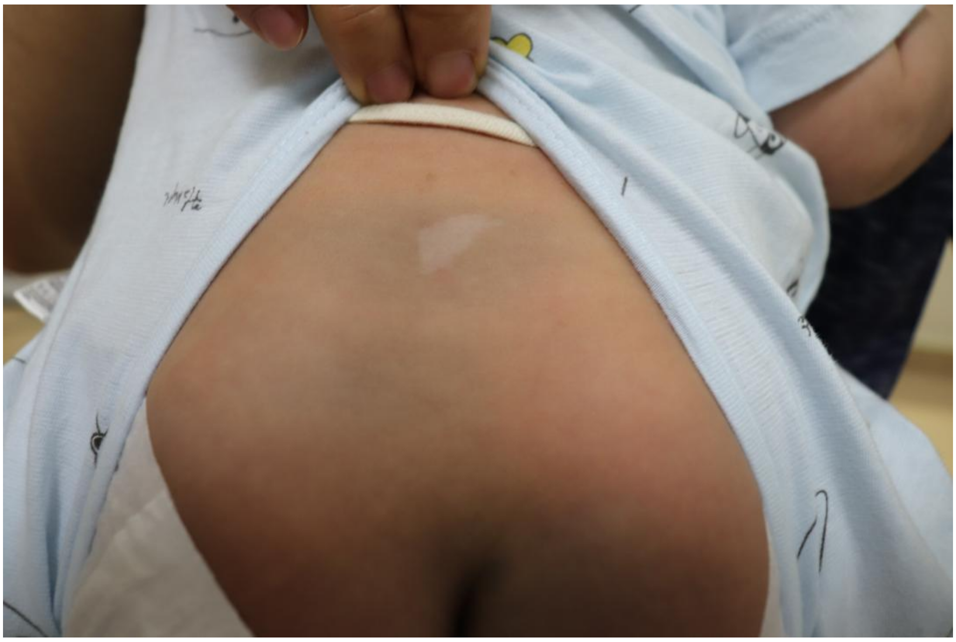

4. A pityriasis versicolor case wrongly classified as vitiligo by 10 out of 14 human raters.

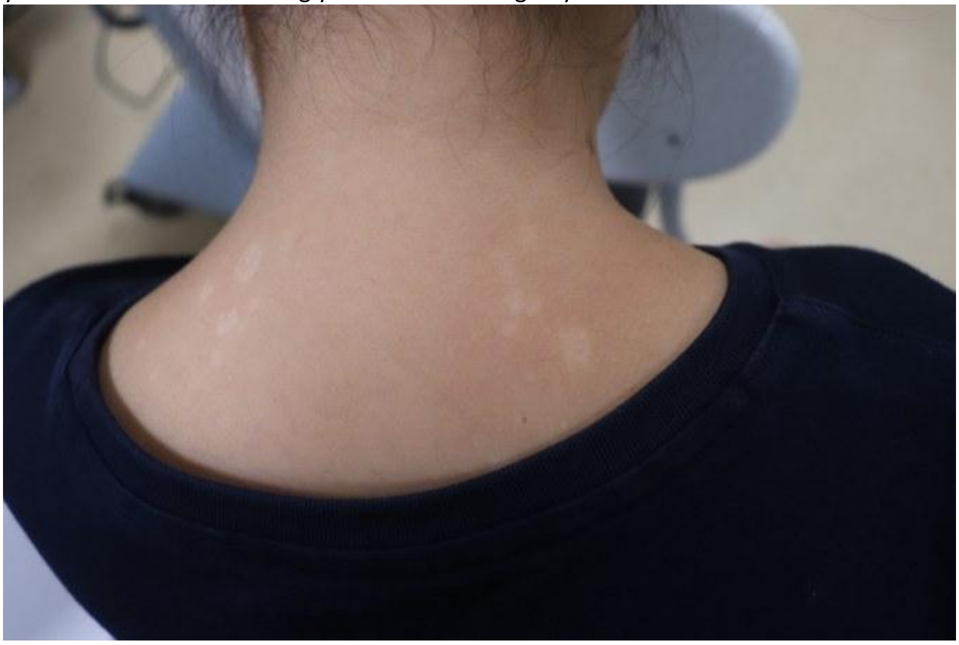

Supplement: Supplementary file 1 [file Presentation_1.PDF]
